# Nivolumab-Induced Colitis in a Patient With Esophageal Adenocarcinoma: A Case Report

**DOI:** 10.7759/cureus.42315

**Published:** 2023-07-23

**Authors:** Iakovos Vlachos, Georgios Karamanolis, Antonios Vezakis, Dionysios Dellaportas, Despoina Myoteri

**Affiliations:** 1 Department of Pathology, Aretaieion University Hospital, School of Medicine, National and Kapodistrian University of Athens, Athens, GRC; 2 Department of Gastroenterology, School of Medicine, National and Kapodistrian University of Athens, Athens, GRC; 3 2nd Department of Surgery, Aretaieion University Hospital, School of Medicine, National and Kapodistrian University of Athens, Athens, GRC; 4 3rd Department of Surgery, Attikon University Hospital, School of Medicine, National and Kapodistrian University of Athens, Athens, GRC

**Keywords:** nivolumab, immune-related adverse events, immune checkpoint inhibitor colitis, immune-mediated colitis, drug-induced colitis

## Abstract

Nivolumab is an immune checkpoint inhibitor used in the treatment of several types of cancer. Among the adverse effects of this drug, immune-mediated colitis (IMC) has been described. However, in contrast to other checkpoint inhibitors, such as ipilimumab, drug-induced colitis due to nivolumab is not commonly reported. We report the case of a 59-year-old male who had undergone surgical resection for gastroesophageal junction adenocarcinoma, had been on nivolumab during the past five months, and presented with worsening diarrhea. Colonoscopy demonstrated local edema and mild colitis in a region of the colonic mucosa located 30 cm distal to the ileocecal valve. Biopsies revealed acute moderate colitis. The patient responded well to loperamide and dietary modifications. Although nivolumab rarely causes IMC, this occurrence requires proper management in order to avoid further complications.

## Introduction

Drug-induced colitis was originally described as a side-effect of nonsteroid anti-inflammatory drugs and is also commonly associated with antibiotic usage. More recently, as immune checkpoint inhibitors have been developed and used in the treatment of several malignancies, immune-mediated colitis (IMC) is reported as a potential drug-induced side effect [1]. These drugs activate certain elements of the immune system to improve antitumor immunity, enhancing the activity of T cells against cancer cells and thus making them a novel treatment option for various types of cancer, such as melanoma, lung cancer, bladder cancer, renal cell carcinoma, and esophageal cancer [2,3]. However, the stimulation of cell-mediated immunity can also target cells of noncancerous tissues; this can result in autoimmune-like/inflammatory side effects, which are collectively termed immune-related adverse events (irAEs). The normal organ systems and tissues that can be affected include the skin, gastrointestinal tract, liver, lungs, and endocrine organs [4]. A case of nivolumab-induced colitis in a patient undergoing adjuvant treatment for esophageal cancer is reported herein, and the importance of this unusual condition is highlighted.

## Case presentation

A 59-year-old Caucasian male with a history of gastroesophageal junction adenocarcinoma treated with neoadjuvant chemotherapy, surgery, and adjuvant chemotherapy, under nivolumab as a second-line oncological treatment, presented with worsening diarrhea. The latter, graded according to the National Cancer Institute’s Common Terminology Criteria for Adverse Events (CTCAE), had started at the fifth month of his treatment with nivolumab, as a grade 1 diarrhea (less than three episodes per day). In the sixth month of therapy, his diarrheal symptoms worsened, with more than five episodes of non-bloody, loperamide-responsive diarrhea per day (grade 2).

**Figure 1:**
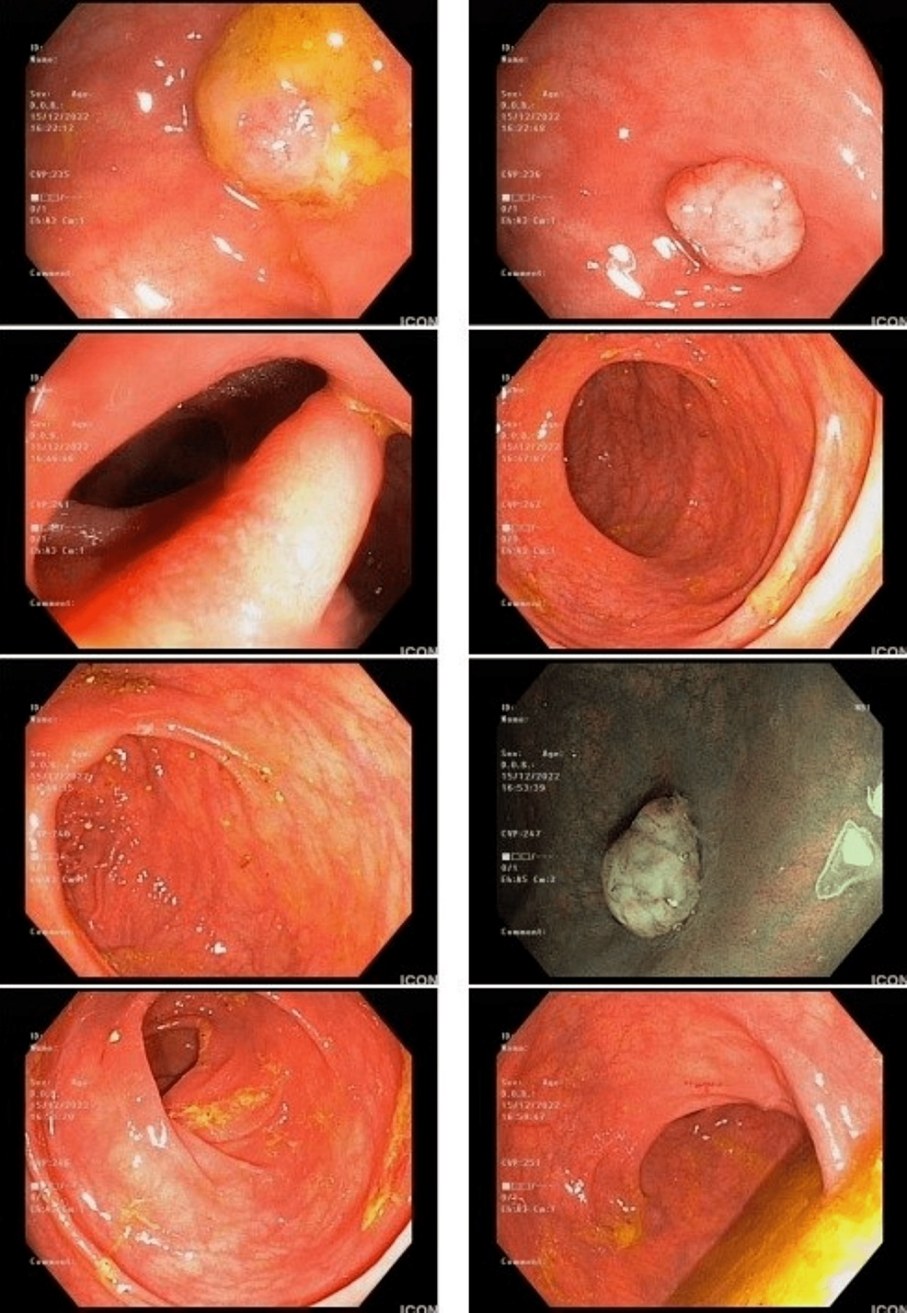
Colonoscopy findings showing a protruding lesion and local edema related to mild colitis

**Figure 2:**
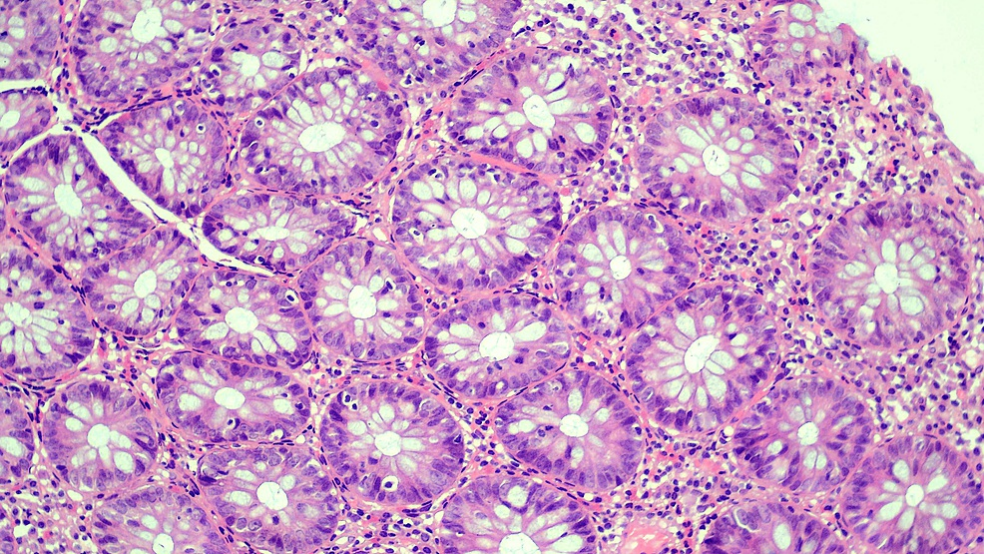
Hematoxylin and eosin staining, demonstrating active and chronic colitis; magnification: 100×

**Figure 3:**
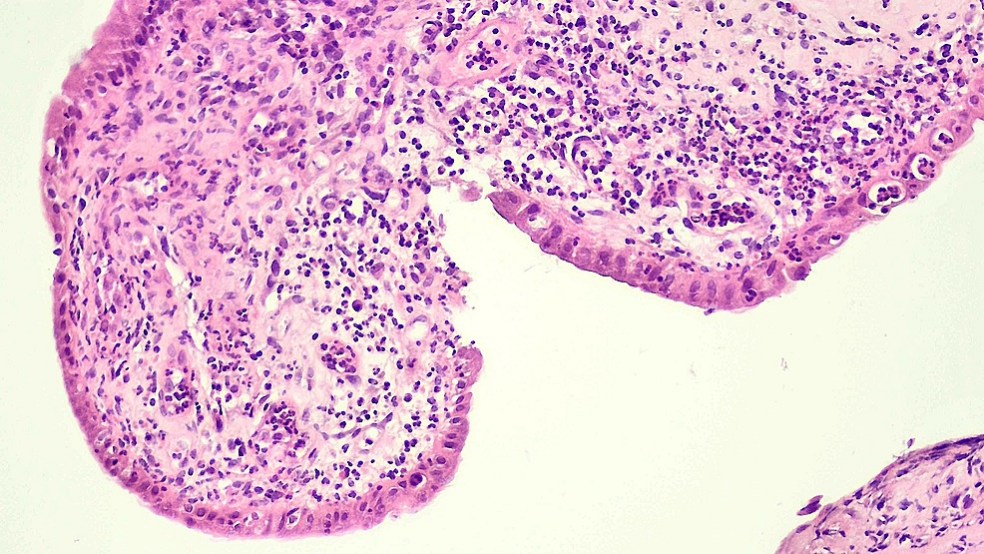
Hematoxylin and eosin staining, showing mucosal erosions and epithelial regenerative changes; magnification: 40×

When experiencing the episodes of diarrhea, the patient reported cramping and generalized abdominal pain. The patient did not experience any other symptoms. Physical examination showed abdominal tenderness. Stool culture and *Clostridium difficile* deoxyribonucleic acid (DNA) amplification stool test were both negative. The patient underwent a colonoscopy, which demonstrated local edema and mild colitis in the ascending colon, approximately 30 cm distal to the ileocecal valve, with a protruding polypoid-like lesion (Figure 1). Several biopsies obtained from the region revealed focal crypt architectural distortion, mucin depletion, and mucosal erosions and ulcerations. In the lamina propria, edema, fibrosis, and moderate inflammatory infiltrate consisting of lymphocytes, plasma cells, eosinophils, and neutrophils were observed. Cryptitis and regenerative changes were also present (Figures 2, 3). Immunostain for *Cytomegalovirus* (CMV) was negative. No granulomas or dysplasias were observed histologically. The patient was treated with loperamide and was ultimately able to complete his six-month treatment cycle with nivolumab.

## Discussion

Immune checkpoint inhibitors are increasingly being used as a treatment option for different types of cancer, including esophageal cancer. IMC is one of the reported adverse events of these drugs. Some of the symptoms of immune checkpoint inhibitor-associated colitis include diarrhea, abdominal pain, hematochezia, fever, and vomiting [1,3,5]. Among these symptoms, diarrhea is the most common but is of variable severity and onset [6]. National Cancer Institute’s Common Terminology Criteria for Adverse Events (CTCAE) has defined the grades of diarrhea and colitis during treatment with immune checkpoint inhibitors. The majority of these cases of diarrhea are mild [1,6]. Specifically, most are categorized as grade 1 (fewer than four stools per day) or grade 2 (four to six stools per day), while grades 3-4 (more than seven stools per day and life-threatening diarrhea, respectively) rarely occur (1%-2% of the patients in a clinical trial with anti-programmed cell death protein 1 {PD-1} therapy) [7,8].

Nivolumab is an intravenously administered fully human IgG4 monoclonal antibody targeting programmed cell death protein 1 (PD-1). Generally, anti-PD-1 drugs are not as toxic compared to standard chemotherapy or other immune checkpoint inhibitors, such as anti-cytotoxic T-lymphocyte-associated protein 4 (CTLA4) drugs (more notably ipilimumab) [9]. Thus, irAEs of anti-PD-1 drugs are neither as frequent nor as severe as those of other immune checkpoint inhibitors [6]. Specifically, colitis induced by ipilimumab has a higher incidence (10%-25%) than nivolumab-induced colitis (1%-5%), while combination therapy increases even more the frequency of colitis (~20%) [10]. Moreover, in contrast to ipilimumab, nivolumab does not seem to have dose-dependent gastrointestinal irAEs, although this must be further studied, as this might be a corollary of the narrow dose spectrum in which nivolumab is typically administered. Finally, it has been reported that 46% of the patients that developed IMC under anti-PD-1 monotherapy subsequently developed additional irAEs during their treatment course, such as hepatotoxicity and skin and endocrine abnormalities. These patients were more likely to experience these adverse events either before (50%) or after (40%) manifesting colitis than concurrently (20%) [5].

The exact pathophysiologic mechanism of immune checkpoint inhibitor-associated colitis is not known. However, some suggested mechanisms include the hyperproliferation and hyperactivation of T cells and lymphocyte infiltration [1].

A detailed history, thorough physical examination, and complete laboratory workup are essential in order to exclude other causes of diarrhea and colitis, such as *Clostridium difficile* or CMV colitis. A colonoscopy is crucial for the diagnosis and must be well-timed. Colonoscopy findings may vary from normal to pancolitis with mucosal ulcerations [11]. Some common colonoscopy findings in patients with IMC are erythema, edema, loss of vascular pattern, erosions, ulcerations, and pseudomembranous colitis. Intestinal superinfections in patients with nivolumab-induced colitis may however lead to the underdiagnosis of the drug-induced colitis [6,9].

Regarding the microscopic findings, there is only a limited number of reports concerning the histologic features of PD-1 inhibitor-associated colitis [6]. As expected, they include edema and the expansion of lamina propria by a mixed inflammatory infiltrate consisting of neutrophils, plasma cells, lymphocytes and eosinophils [1,6,7]. Other findings include crypt architectural distortion, neutrophilic crypt abscesses, and features of ischemic or collagenous colitis [6]. Rarely, granulomas can be observed, usually in association with crypt rupture. Recently, it has been suggested that the detection of apoptotic bodies could be a feature of immune-mediated colitis [6,7,9], since PD-1 inhibitor-associated colitis often exhibits active colitis with increased apoptosis, while an increase in the number of intraepithelial lymphocytes has been observed only in a small number of cases. Up to date, two major patterns of anti-PD-1-induced colitis have been described. The first and most common pattern consists of active colitis along with increased crypt epithelial cell apoptosis and crypt atrophy/dropout. Microscopically, the active colitis consists of intraepithelial neutrophils and neutrophilic cryptitis with the formation of neutrophilic crypt microabscesses. The second and less common pattern of anti-PD-1-mediated colitis was characterized by a lymphocytic colitis similar to the one seen in anti-CTLA4-induced colitis [6].

The diagnosis and proper treatment of immune-mediated colitis are pivotal for the patient, as several cases of intestinal perforation due to colitis after treatment with immune checkpoint inhibitors have been reported [5,6]. Generally, the optimal management of any adverse effect of immune checkpoint inhibitors is prompt recognition; supportive treatment; and, depending on the severity of the illness and the patient’s response to supportive measures, the administration of an immunosuppressive agent such as corticosteroids or anti-tumor necrosis factor-alpha (TNF-α). Specifically, the proper steps for the management of immune-mediated colitis depend on the grading of the diarrhea. Hence, grade 1 or 2 diarrhea is treated with an anti-diarrheal diet, hydration and electrolyte supplementation, and the administration of loperamide, atropine sulfate, or diphenoxylate hydrochloride. In cases of persistent grade 2 diarrhea (defined as four to six stools per day for more than three days), steroids are initiated. For grade 3-4 diarrhea, the confirmation of colitis is needed. This can be done by colonoscopy or abdominal computed tomography. The management of grade 3-4 diarrhea includes parenteral hydration, nutritional support, and early intravenous corticosteroid administration, followed by high doses of oral corticosteroids. In cases that the patient does not show signs of improvement, treatment with infliximab may be considered. For severe toxicity, such as in cases of peritoneal signs consistent with bowel perforation, ileus, or fever, immune checkpoint inhibitor treatment should be permanently discontinued and surgical consultation sought [6,7].

The case presented above underlines that nivolumab monotherapy can induce IMC. To the best of our knowledge, such a reaction has not been reported before in an esophageal adenocarcinoma patient.

## Conclusions

Due to the increasing use of immune checkpoint inhibitors, it is reasonable to expect that immune-mediated adverse effects in the gastrointestinal tract will become more frequent. So, in patients with the symptoms of worsening colitis and diarrhea, after infectious causes are excluded, a colonoscopy should be performed and colonic biopsies obtained. Although anti-PD-1-induced colitis constitutes a rare adverse event, proper workup is needed to avoid potentially life-threatening complications.
